# African Americans, Caribbean Blacks and Depression: Which Biopsychosocial Factors Should Social Workers Focus On? Results from the National Survey of American Life (NSAL)

**DOI:** 10.1007/s10597-021-00833-6

**Published:** 2021-05-07

**Authors:** Michael A. Robinson, Irang Kim, Orion Mowbray, Lindsey Disney

**Affiliations:** 1 School of Social Work, University of Georgia, 279 Williams St., Athens, GA 30602 USA; 2 Tulane University, New Orleans, LA 70112 USA; 3 The University at Albany, 1400 Washington Ave, Albany, NY 12222 USA

**Keywords:** Depression, African Americans, Caribbean Blacks, National Survey of American Life (NSAL), Biopsychosocial, Treatment, Prevention, Social workers

## Abstract

Research suggests that African Americans may be more likely to experience depression, especially severe depression, than other racial or ethnic groups in the United States. Overall there is scant research comparing the relationship between ethnicity and depression among the U.S. Black population. The purpose of this study is to identify the most significant biopsychosocial factors social workers can address in the prevention and treatment of depression in African American and first generations Caribbean Black clients. Data was from the National Survey of American Life (NSAL). Bivariate associations showed that respondents who reported higher self-esteem, lower hopelessness, higher sense of mastery, and lower discrimination showed lower likelihood of having Major Depressive Disorder (MDD). The logistic regression model suggested that respondents who have ever had a chronic disease were more likely to report depression than those who have not ever had a chronic disease. Caribbean Blacks were more likely to report depression compared to African Americans. Additionally, respondents who reported higher discrimination scores were more likely to report depression. This study suggests that social workers should embrace the interconnectedness and holistic approach of the biopsychosocial model in their case conceptualizations, prevention strategies, and treatment modalities.

## Introduction

### African Americans and Depression

Current research suggests that African Americans may be more likely to experience depression, especially severe depression, than other racial or ethnic groups in the United States (Bailey et al., 2019; CDC, 2010; Williams, Gonzalez, et al., 2007; Williams, Norman, et al., 2007). Survey data collected by the CDC (2010) found that 13% of African Americans met the criteria for any depression compared to 8% of non-Latino whites and 11% of Latinos. Additionally, African Americans are more likely to experience persistent depression than non-Latino whites and less likely to obtain treatment for their depression (Williams, Gonzalez, et al., 2007; Williams, Norman, et al., 2007). This inconsistency between the prevalence of depression within the community and the underutilization of services by African Americans has been attributed to barriers experienced by African American individuals as well as relevant cultural factors. The experience of discrimination and prejudice is not only a significant risk factor for the onset of depression among African Americans (Britt-Spells et al., 2018), but it also represents a structural barrier that precludes many African Americans from obtaining mental health care. Moreover, individuals who belong to marginalized racial and ethnic groups often have unfavorable attitudes towards mental health services that affect whether or not they seek treatment (Watson & Hunter, 2015).

### Caribbean Blacks and Depression

Both^1^Caribbean Blacks and African-Americans are included within the same racial category. Despite this, there are some differences between the two populations with regards to protective factors for depression (Molina & James, 2016). Research has found that Caribbean Blacks generally belong to a higher socioeconomic status and may be immigrants to the United States (Mouzon & McLean, 2017; Molina & James, 2016), both of which are protective factors against mental illness (Hastings & Snowden, 2019). Despite this, some studies have reported that Caribbean Blacks report higher prevalence rates of depression and depression symptoms when compared to African Americans. These findings have been partially attributed to research that shows there is no difference between African Americans and Caribbean Blacks living in the U.S. in perceived discrimination (Marshall & Rue, 2012). Interestingly researchers have found that Caribbean Blacks adolescents whose parents were immigrants reported fewer depressive symptoms than whose parents were born in the U.S. (Cullins et al., 2019; Smith, 2019).

Furthermore, despite differences in background African-Americans and Caribbean Blacks have similar experiences with prejudice and discrimination and these experiences are positively associated with depressive symptoms for both groups (Marshall & Rue, 2012). For example, research has shown a significant, positive relationship exists between experiences of everyday racism and past-year Major Depressive Disorder for both African-Americans and Caribbean Blacks (Molina & James, 2016). Even so, the same study found that higher levels of internalized racism were associated with decreased risk of past-year MDD, but only for participants who were Caribbean Blacks (Molina & James, 2016). These results suggest that this population may engage in functional internalization of stereotypes that may serve as a self-protective factor against mental illness (Molina & James, 2016). For example:

For example, one study found that women who failed a math test but were reminded of negative stereotypes tied to women and math performance rated their self-esteem higher than those who were not reminded of the negative stereotypes; and endorsement of these negative stereotypes were greater among women with higher levels of self-esteem compared to those with lower self-esteem (Burkley & Blanton, 2008, p. 1)

## Theoretical Model

In an effort to better understand the factors that influence depression in African Americans and Caribbean Blacks, the authors have adapted the biopsychosocial model (Engel, 1977), a model rooted in systems theory which has been explored as a framework for studying mental and physical disorders (Schwartz, 1982; Dodge & Petitt, 2003; Ricciardelli & McCabe, 2004; Buckner et al., 2013). The biopsychosocial model espouses that there are biological, psychological, and social factors that contribute to health outcomes (Engles, 1977). The model illustrates how “physical health and well being are shaped by the interactions between biological, psychological, and social factors” (Suls & Rothman, 2004, p. 121). Suls and Rothman (2004) go on to say that the full potential of the biopsychosocial model is untapped. The literature on using the biopsychosocial model as a framework for exploring depression in African Americans is scant; however, Di Benedetto (2010) have used the model as a framework for examining depression in patients with Acute Coronary Syndrome (ACS). They describe the biological and social factors associated with depression in patients with ACS as age, gender, education, income, stays in hospital, hypertension, diabetes mellitus, smoking, exercise, perceived social support, social isolation, employment status, and previously reported depression. Di Beneditto and associates (2010) further describe psychological factors that influence depression as stress and social, physical, spiritual, and cognitive coping skills. On the other hand, Suls and Rothman (2004) point out that, “… the degree to which the biopsychosocial model has been embraced by the biomedical establishment is unclear” (p. 120), and they further argue that the biopsychosocial model is a *work in progress.*

## Biopsychosocial Factors Influencing Depression

### Biological Factors

There is a large body of research that revealed a relationship between age and depression (Brown et al., 1995; Beard et al., 2009; Wiber et al., 2009; Singh, 2015). “The prevalence of depression is high among elderly persons, and longitudinal studies have found modest increases in depressive symptoms with age” (Beard et al., 2009, p. 1308). Wiber et al. (2009) found that African Americans experience higher symptom levels in early adulthood in comparison to whites, but equivalent levels by middle age. However, other studies have shown that in conjunction with age there are other factors that influence depression such as life’s events, for example, marriage, birth of children, death of parents, spouse, and children, retirement… (Mirowski & Ross, 1992; Santini et al., 2015). Research revealed that there is strong association between gender and depression in that females have a higher prevalence than males and it has been widely documented that there are gender difference in depression prevalence, with women experiencing major depression about twice as often as men (Brown et al., 1995; Martin et al., 2013; Schuch et al., 2014).

Comorbidity is another important element affecting mental health whereas comorbid diseases may have unexpected yet clinically significant effects on patients' health (Chapman et al., 2005; Voaklander et al., 2004; Xuan et al., 1999). Furthermore, people with chronic illnesses have higher prevalence rates of depression (Katon & Ciechanowski, 2002; Verma et al., 2017). Likewise, researchers have found that depressive disorders influence the severity of physical illnesses such as coronary artery disease, diabetes mellitus, hypertension, myocardial infarction, heart disease, chronic pain, chronic obstructive pulmonary disease (COPD), obesity, and cancer (Chapman and associates, 2005; Casssano & Fava, 2002; Katon & Ciechanowski, 2002; Verma et al., 2017).

### Psychological Factors

The psychological vulnerabilities and strengths which can exacerbate or mitigate psychiatric symptomatology**.** The first is Mastery, which is widely used to discern patterns of self efficacy is also an important indicator of depression. Several studies have found a significant negative correlation between mastery and depression (Schreiner & Morimoto, 2003). Chung et al. (2007) found that higher levels of mastery result in a higher quality of life which affects the level of depression experienced by the individual. Basically, the higher the mastery, the lower the level of depression experienced. According to the National Institutes of Mental Health (NIMH) (2017), long-term stress can increase the risk of diseases like depression, heart disease, and a variety of other problems. Moreover, the Diathesis Stress Model posits, “…stress may activate a diathesis or vulnerability, transforming the potential of predisposition into the actuality of psychopathology.” (Colodro-Conde et al., 2018 p. 1590). In other words stress has a propound impact on mental health.

The relationship between self-esteem and depression has been examined conceptually and empirically in many studies. Self-esteem has been found to be a central component of depressive symptoms as well as being negatively correlated. Moreover, low self-esteem plays a decisive role in the onset of depression (Beck, 1967; Inkson, 1978; Brockner & Guare, 1983; Tennen & Herzberger, 1987; Sowislo & Orth, 2013; Steiger et al., 2014; Rieger et al., 2016). In a study by Maestas et al. (2008) using a sample of 24 women with high depression and 28 women with low depression, they found significant differences between low depression–vulnerable and high depression–vulnerable women’s level of self–esteem. Many studies have documented the strong negative correlation between self-esteem and depression (Brockner & Guare, 1983; Tennen & Herzberger, 1987; Schöne et al., 2014). Lastly, hopelessness has been depicted in many studies as one of the indicators of depression (Murphy et al., 2000; Sharma & Sinha, 2015), and theories of depression have also indicated that hopelessness is a key characteristic (Beck, 1967; Beck et al., 1979). Ceylan and Aral (2007), they found a significant correlation between hopelessness and depression.

### Social Factors

Williams, Gonzalez, et al. (2007), Williams, Norman, et al. (2007)) found that educational attainment influences prevalence rates for depression. They revealed that from 0 – 11 years of education, non-Hispanic whites (12.4%) had higher prevalence rates than African Americans (11.3%); and at 12 years of education, non-Hispanic whites (17.1%) had the highest lifetime prevalence rate, than African Americans (9.1%). Research has shown that income figures prominently into prevalence rates for depression and Williams et al., (2008) found that non-Hispanic whites have the highest lifetime prevalence rate of MDD by the various income levels examined; however, there is no significant difference by income. On the other hand, Santiago et al. (2011) disagree, they found that lower income levels figure prominently into higher levels of depression. Religion, for the purpose of this research is an organized system of beliefs, practices, rituals, and symbols. Spirituality is a quest for understanding of the meaning of life with a higher power of one’s own choosing. Wittink et al. (2009) found that there is research linking religious involvement with psychological well-being among African Americans, and it indicates that prayer is an important means of coping with serious personal problems. Williams, Gonzalez, et al. (2007), Williams, Norman, et al. (2007)) found that by race, African Americans have lower lifetime prevalence but a higher risk of the persistence of MDD than non-Latino whites. Marital status was examined by Woodward et al., (2008) and they found that individual who were previously married and never married sought mental health services more than those who were married. Additionally, according to Frech and Williams (2007) there is a growing body of research literature suggesting that married couples experience fewer symptoms of depression, less stress, better health, and a higher sense of well-being than couples who are unmarried. The relationship between perceived social support and depression has been examined and researchers revealed that there is an inverse relationship. The more perceived social support the lower the levels of depression (Guerette & Smedema, 2011).

Recent studies have shown that there is a relationship between discrimination and depression. Schulz and et al. (2006) found a causal relationship that showed evidence that a change over time in everyday discrimination is associated with a change over time in symptoms of depression. Discrimination may affect an individual’s sense of control and promote hopelessness and these factors in turn may lead to depression or another mental disorder (Williams & Williams-Morris, 2000; Perlow et al., 2004; English et al., 2014; Kolarcik et al., 2015). In a national study exploring mental health and depression, the researchers found that minorities who were discriminated against had poorer health outcomes than non-Latino whites (Roberts et al., 2004) and Borrell and et al. (2006) found their study on self reported physical and mental health, racial discrimination and skin color that there is a statistically significant relationship between discrimination and mental health. Clearly, there are numerous biopsychosocial factors that influence depression in African Americans; however, this study is investigating the most significant factors social workers can address in the treatment of depression in African American males.

## Method

### Sample

The NSAL yielded 6199 adult interviews with eighty-six percent completing face to face with participants who self identified in the following three categories: African American, Afro-Caribbean, and non-Latino whites. For the purposes of this research, we will examine 332 African American and Caribbean Blacks. The NSAL is the largest dataset to date on mental health and African Americans and was collected from a representative sample of the US adult population. The strength of this national dataset is the sufficient power to investigate cultural and ethnic influences on mental disorders. Taking into account that there are conceivably hundreds of biological and psychosocial factors, the authors have elected to use factors present in the NSAL dataset for the current study in an effort to better understand the factors that influence depression in African Americans from a biopsychosocial perspective.

### Measures

#### Chronic Illness

Chronic illness was assessed through participant responses to four chronic diseases; diabetes, stroke, cancer, and heart disease. The selection of specific chronic disease was based on Centers for Disease Control guidelines (Center for Disease Control, 2021). Participants were asked (1) Has a professional ever said you have heart trouble? (2) Has a professional ever said you have cancer? (3) Has a professional ever said you had a stroke? (4) Has a professional ever said you have diabetes? Participant responses were dichotomized where a yes response to at least one of these questions was considered to be an indicator of a chronic illness.

#### Family History of Depression

Family history of depression was measured by participant responses to a question that examined the number of close relatives with depression. This count-based measure was dichotomized and participants who reported at least one close relative with depression were considered to have family history of depression.

#### Self-Esteem

Participants in the NSAL completed the Rosenberg self-esteem scale (Rosenberg, 1965). The scale is comprised of 10 items (e.g., “I am person of worth/equal to others” and “I do things as well as others”) with a four response scale (1 = strongly disagree, 2 = disagree, 3 = agree, and 4 = strongly agree). Participant responses were scaled to create a sum self-report item (α = 0.59).

#### Hopelessness

NSAL participants completed the Everson Hopelessness Scale (Everson, et al., 1996), This scale is comprised of two items (1) I sometimes think I am no good, and (2) It is impossible to reach my goals with a four response scale (1 = strongly disagree, 2 = disagree, 3 = agree, and 4 = strongly agree). These items were summed together and recoded where participant responses of less than five indicated moderate hopelessness and participant responses above six indicated high hopelessness (Everson et al., 1996).

#### Sense of Mastery

Sense of mastery was measured by Mastery scale (Pearlin, 1989), which comprised seven items (e.g., “Can do just about anything set my mind to”, “What happens to me in future depends on me”) with a four response scale (1 = strongly disagree, 2 = disagree, 3 = agree, and 4 = strongly agree). Participant responses were scaled to create a sum sense of mastery item (α = 0.72).

#### Spirituality

Spirituality was assessed by a single question ‘how spiritual are you?’ along a four-point response scale (1 = not spiritual at all, 2 = not too spiritual, 3 = fairly spiritual, 4 = very spiritual).

#### Social Support

Social support was measured by three items asking frequency of helping out from family, friends, and church members with a four response scale (1 = never, 2 = not too often, 3 = fairly often, and 4 = very often). Participant responses were scaled to create a sum social support item (α = 0.55).

#### Discrimination

Respondents completed extended version of the everyday discrimination scale (Williams et al., 1997) comprising of 10 items with a six-point scale (1 = Never, 2 = Less than once a year, 3 = A few times a year, 4 = A few times a month, 5 = At least once a week, 6 = Almost everyday). Participant responses were scaled to create a sum social support item (α = 0.89).

#### Depression

To assess past-year DSM-IV Major Depressive Disorder, a dichotomous variable indicated whether respondent have had Major Depressive Disorder within 12 months.

### Analytic Strategy

STATA Version 12 (Statacorp, 2011) were used to complete all analysis. Sample characteristics and bivariate analyses were examined for all measured biopsychosocial variables described above. Bivariate associations examined associations between Major Depressive Disorder and biopsychosocial variables. A multivariate logistic regression model was used to examine which biopsychosocial variables affect Major Depressive Disorder. Analysis incorporated the survey weighting design provided in NSAL.

## Results

### Sample Characteristics and Bivariate Associations

Overall, 5.6% of the entire sample reported they endorsed 12 months Major Depressive Disorder. Table 1 shows a summary of sample characteristics and bivariate associations with MDD. About 17% of respondents reported that they have had one of the chronic diseases. 22.24% of respondents reported at least one close relative who had depression. Mean of ages is 39.54. Self-esteem ranges from 0 to 40 (M = 32.45). Hopelessness ranges from 0 to 8 (M = 3.23). Sense of mastery ranges from 0 to 24 (M = 22.31). About 23.90 of respondents completed less than 11 years of education. The largest percentage of reported income was between $35,000- $69,999. About 73% of respondents were employed. Mean of spirituality is 3.22 which corresponded with the “fairly spiritual.” Social support ranges from 0 to 12 (M = 7.93) and discrimination ranges from 0 to 60 (M = 25.48).

**Table 1:** Sample descriptive and bivariate associations with major depressive disorder

	Overall % N = 332 % (M)	MDD % (M)	*t (F)*
Chronic disease^a^			3.09
Chronic disease	16.97	4.82	
No chronic disease	83.03	2.88	
Family history for depression			7.22**
Family history for depression	22.24	5.10	
No family history for depression	77.76	2.35	
Erthnicity			.004
Caribbean Black	30.66	3.27	
African American	69.34	3.20	
Age			.381
18 to 24	15.22	3.65	
25 to 34	19.15	3.21	
35 to 54	42.99	3.29	
55 and over	22.64	2.81	
Self-esteem	(32.45)	(30.00)	67.56 (−.185)**
Hopelessness	(3.23)	(4.19)	12.92 (.25)**
Mastery	(22.31)	(20.14)	26.92 (−.15)**
Education			2.04
0–11 years	23.90	3.34	
12 years	36.55	3.72	
13 – 15 years	23.19	2.17	
Greater than 16 years	16.37	3.45	
Income^b^			4.26
0 – 9,999	11.02	5.56	
10,000 – 19,999	16.37	3.10	
20,000 – 34,999	22.95	3.15	
35,000 – 69,999	34.32	2.95	
70,000 or more	14.35	2.34	
Work			3.29
Employed	72.26	2.81	
Unemployed	8.37	5.37	
Not in labor force	19.37	3.83	
Spirituality^c^	(3.22)	(3.16)	.38 (−.11)
Social support	(7.93)	(7.40)	1.74 (−.06)
Discrimination^d^	(25.48)	(30.21)	17.06 (.06)**

Numbers in column headings represent unweighted values. All analyses incorporate the NSAL sample weighting strategy

p < 05, **p < .01

^a^Measured as the presence of heart disease, cancer, stroke or diabetes

^b^Measured as annual household income

^c^4 point scale 1 = Not spiritual at all, 2 = Not too spiritual, 3 = Fairly spiritual, 4 = Very spiritual

^d^6 point scale 1 = Never, 2 = Less than once a year, 3 = A few times a year, 4 = A few times a month, 5 = At least once a week, 6 = Almost everyday

Bivariate associations showed that those who have a family history of depression (M = 5.10) reported significantly higher MDD than those who do not have a family history of depression (M = 2.35), X^2^ (1, N = 332) = 7.22, p < 0.01. Respondents who reported higher self-esteem, lower hopelessness, higher sense of mastery, and lower discrimination showed lower likelihood of having MDD.

### Multivariate Model Predicting Depression

Table 2 presents multivariate logistic regression model of depression displaying odds ratio and 95% of confidence interval for each biopsychosocial variable. Model results showed that respondents who have ever had chronic disease were more likely to report depression than those who have not ever had chronic disease (OR = 2.65, *p* < 0.05). Caribbean Blacks were more likely to report depression compared to African Americans (OR = 0.28, *p* < 0.01). Respondents who reported lower self-esteem were more likely to show depression (OR = 0.88, *p* < 0.01). Respondents who reported higher discrimination were more likely to show depression (OR = 1.05, *p* < 0.05). Family history of depression, age, hopelessness, sense of mastery, education, income, employment status, spirituality, and social support were not significantly associated with depression.

**Table 2:** Multiple logistic regression model of depression

	Depression^a^ (N = 332)	
	OR	95% CI
Biological factor		
Chronic disease	2.65*	[1.06, 6.64]
Family history for depression	1.49	[.558, 4.00]
Ethnicity (African American)	.285**	[.134, .609]
Age		
18 to 24	–	
25 to 34	.685	[.225, 2.09]
35 to 54	.541	[.192, 1.53]
55 and over	.468	[.115, 1.91]
Psychological factor		
Self-esteem	.883*	[.793, .983]
Hopelessness	1.02	[.833, 1.24]
Mastery	.968	[.839, 1.12]
Social factor		
Education		
0 –11 years	–	–
12 years	.756	[.229, 2.49]
13 – 15 years	.607	[.171, 2.15]
Greater than 16 years	.825	[.173, 3.92]
Income^b^		
0 – 9,999	–	–
10,000 – 19,999	.735	[.245, 2.20]
20,000 – 34,999	1.16	[.377, 3.55]
35,000 – 69,999	1.32	[.360, 4.87]
70,000 or more	1.49	[.517, 4.34]
Work		
Employed	–	–
Unemployed	1.93	[.525, 7.08]
Not in labor force	1.07	[.362, 3.18]
Spirituality^c^	1.07	[.734, 1.55]
Social support	.961	[.854, 1.08]
Discrimination	1.05*	[1.01, 1.10]

Numbers in column headings represent unweighted values. All analyses incorporate the NSAL sample weighting strategy

*p < 05, **p < .01

^a^Measured as the presence of heart disease, cancer, stroke or diabetes

^b^Measured as annual household income

^c^4 point scale 1 = Not spiritual at all, 2 = Not too spiritual, 3 = Fairly spiritual, 4 = Very spiritual

## Discussion/Implications

The results from this study give direction to social workers as to which biopsychosocial factors may have the strongest impact on depression in African Americans and Caribbean Blacks. Bivariate correlations, as well as a plethora of other research, find that the biopsychosocial variables of family history of depression, self-esteem, hopelessness, a sense of mastery, and discrimination all have a significant relationship with one’s mental health. However, while these biopsychosocial variables were found to have a correlational relationship with depression, they were not all found to be significant predictors of depression once they were put into a multivariate model. This study suggests that when other variables are controlled for, the biopsychosocial variables that remain significant predictors of MDD are chronic disease, self-esteem, ethnic group identity, and experiences of discrimination. Thus, one logical implication for social workers is that in order to have maximum impact, social work efforts may want to focus on addressing these four most significant areas of concern.

Additionally, this study supports the biopsychosocial model, and is supported by the biopsychosocial model. The four significant multivariate findings are across the biopsychosocial sphere – with chronic disease being a biological factor, self-esteem being a psychological factor, and ethnic identity and discrimination being social factors. This study suggests that social workers should embrace the interconnectedness and holistic approach of the biopsychosocial model in their case conceptualizations, prevention strategies, and treatment modalities.

### Chronic Disease Comorbidity

This study adds to the body of current literature on the prevailing higher rates of depression for those living with chronic illnesses (Katon & Ciechanowski, 2002; Verma et al., 2017). Chronic diseases are a biological factor, which, according to this study, has a strong ability to predict who is at risk for MDD. Social workers who have clients with coexisting physical health and mental health issues should utilize a holistic treatment plan to address both areas of concern. For example, a holistic mental health treatment plan could include not only psychological treatment, but also case management services for managing chronic diseases (i.e., assistance with arranging multiple doctor appointments, attending appointments, filling prescriptions, implementing a daily medication regime, or advocating for pro bono services).

For African Americans and Caribbean Blacks, racial inequity in the healthcare system may result in chronic diseases having more of a negative impact on other facets of life, when compared to their white counterparts. Of course, racial inequity is directly tied to discrimination, which was another significant predictor of MDD in this study. Systematic discrimination can affect access to medical care, and conscious or subconscious discrimination from healthcare providers can affect quality of medical care (Williams & Mohammed, 2009). Social workers at the policy level should continue to advocate for affordable healthcare or other policy interventions that target racial inequity in healthcare (Copeland, 2005; Williams & Jackson, 2005). Social work practitioners in the healthcare field should understand their own implicit bias, and can help change to occur by providing trainings on racial inequity for medical staff (Burgess et al., 2007), or by advocating for clients who may perceive discrimination in their medical care.

### Self-Esteem

This study found that self-esteem, conceptualized as a psychological factor in one’s life, was a significant predictor of MDD. Given that feelings of worthlessness are an inherent part of depression, and a criterion for a MDD diagnosis, this is not surprising. There is a large body of evidence that self-esteem is interconnected with depression, as well as with chronic disease (Juth et al., 2008), ethnic identity (Smith & Silva, 2011), and discrimination (Thoits, 2010). In order to most effectively address self-esteem, it may be helpful to explore with clients what are the possible underlying sources negatively affecting their feelings about themselves. For example, for clients with chronic illnesses, self-esteem may need to be addressed in the larger context of self-care (Juth et al., 2008). Meanwhile, for clients who identify discrimination as a root cause of low self-esteem, social workers may want to focus on empowerment and coping interventions.

### Ethnic Group Identity

One interesting finding from this study was that Caribbean Blacks had a higher association with depression than African Americans. Previous research comparing depression between Caribbean Blacks and African Americans has been mixed, and overall, there is scant research comparing the relationship between ethnicity and depression among the U.S. Black population (Williams, Gonzalez, et al., 2007; Williams, Norman, et al., 2007). The reason this finding is surprising is twofold: (1) African Americans have more risk factors for depression and are also more likely to report depression than other ethnic groups (CDC, 2010) *and* (2) Caribbean Blacks have more protective factors against depression (Molina & James, 2016), for example, higher levels of household income and education (Baily et al. (2019). Thus, one might logically – but, perhaps, wrongly—presume that African Americans are more at risk for depression than Caribbean Blacks.

Research is needed to further understand differences among African Americans and Caribbean Blacks, in order to know who is most at risk for experiencing MDD within racial subgroups and where to target prevention measures. One possible explanation for why Caribbean Blacks may have higher rates of MDD than African Americans is that Caribbean Blacks are less likely to seek treatment (Williams, Gonzalez, et al., 2007; Williams, Norman, et al., 2007). Social workers working should keep in mind that mental health stigma may be high among Caribbean Black clients and building rapport to talk about depression may take more time than with other Black clients. Additionally, social workers should be mindful that there is much heterogeneity within any racial group, and given the lack of ample knowledge about subgroup differences among African Americans and Caribbean Blacks, adequately assess each individual client.

### Discrimination

Overall, this study also adds to the body of discrimination research that finds a predictive link between discrimination and mental illness (Borrell et al., 2006; Roberts et al., 2004). Clinical social workers should be mindful of the U.S. historical context and current sociopolitical climate that African Americans are living in, and how discrimination experiences may be negatively affecting mental health. Further, social work practitioners should probe for such possible experiences, rather than assuming the client will share. This practice recommendation is especially relevant for clinical social workers who, based on their own ethnic or other identities, may have more social privileges and no or few personal experiences of discrimination from which to draw reference. Treatment plans for African Americans and Caribbean Black clients who are depressed may need to include the development of coping skills for discrimination and oppression. Clinical social workers should also incorporate a reflexive practice of examining ways in which they may be unintentionally perpetuating bias and discrimination in their clinical work, such as through discriminatory case conceptualizations or biased diagnoses. Similarly, social work educators should teach students about the many forms of racial discrimination, such as explicit discrimination, subconscious discrimination, implicit bias, microassaults, microinsults, microinvalidations, profiling, stereotyping, and structural racism (Jackson & Samuels, 2011).

Given the connection between racial discrimination and mental illness, there is a need for social workers to engage in anti-racism activism, such as through policy reform and community organizing. The social work profession cannot focus solely on individual level interventions but instead must engage in the larger structures that perpetuate systematic racism. While this study examined individual discrimination experiences, further research should examine the impact of historical and community level discrimination on depression.

Lastly, it is important to point out that although minority racial identities may put individuals at risk for discrimination, and, thus, increased risk for depression, the literature shows that, overall, racio-ethnic identity has more positive effects on well-being than negative effects (Smith & Silva, 2011; Thoits, 2010). Thus, social workers should utilize a strengths perspective and empowerment theory to support a client’s racial identity since this serves as a protective factor against discrimination. Moreover, this study highlights the utility of the biopsychosocial model in identifying factors that social workers can address when working with African Americans and Caribbean Blacks suffering with depression.

## Footnotes

1 Please note that this paper is referencing first generation Caribbean Blacks.
