# Correction: African Americans, Caribbean Blacks and Depression: Which Biopsychosocial Factors Should Social Workers Focus On? Results from the National Survey of American Life (NSAL)

**DOI:** 10.1007/s10597-024-01229-y

**Published:** 2024-01-11

**Authors:** Michael A. Robinson, Irang Kim, Orion Mowbray, Lindsey Disney

**Affiliations:** 1 School of Social Work, University of Georgia, 279 Williams St., Athens, GA 30602 USA; 2 Tulane University, New Orleans, LA 70112 USA; 3 The University at Albany, 1400 Washington Ave, Albany, NY 12222 USA

**Correction to: Community Mental Health Journal (2022) 58:366–375** 10.1007/s10597-021-00833-6

The article ‘‘African Americans, Caribbean Blacks and Depression: Which Biopsychosocial Factors Should Social Workers Focus On? Results from the National Survey of American Life (NSAL)”, written by Michael A. Robinson, Irang Kim, Orion Mowbray and Lindsey Disney was originally published electronically on the publisher’s internet portal on 7 May 2021 without open access. With the author(s) decision to opt for Open Choice, the copyright of the article changed on 19 December 2023 to The Author(s) 2021 and the article is forthwith distributed under a Creative Commons Attribution 4.0 International License, which permits use, sharing, adaptation, distribution and reproduction in any medium or format, as long as you give appropriate credit to the original author(s) and the source, provide a link to the Creative Commons licence and indicate if changes were made. The images or other third-party materials in this article are included in the articles Creative Commons licence, unless indicated otherwise in a credit line to the material. If material is not included in the articles Creative Commons licence and your intended use is not permitted by statutory regulation or exceeds the permitted use, you will need to obtain permission directly from the copyright holder. To view a copy of this licence, visit http://creativecommons.org/licenses/by/4.0.

